# Reserve Accumulation Is Prioritized Over Growth Following Single or Combined Injuries in Three Common North American Urban Tree Species

**DOI:** 10.3389/fpls.2021.715399

**Published:** 2021-08-06

**Authors:** Jorge Andres Ramirez, Valentina Vitali, Jordi Martínez-Vilalta, I. Tanya Handa, Christian Messier

**Affiliations:** ^1^Facultad de Ciencias Agrarias, Universidad del Cauca, Popayán, Colombia; ^2^Centre d’Étude de la Forêt (CEF), Université du Québec à Montréal, Montreal, QC, Canada; ^3^WSL Research Unit Forest Dynamics, Research Group Ecosystem-Ecology Swiss Federal Institute for Forest, Snow and Landscape Research WSL, Birmensdorf, Switzerland; ^4^CREAF, Universitat Autònoma de Barcelona, Bellaterra, Spain; ^5^Faculté des Sciences, Département des Sciences Biologiques, Université du Québec à Montréal, Montreal, QC, Canada; ^6^Institut des Sciences de la Foret Tempérée, Université du Québec en Outaouais, Ripon, QC, Canada

**Keywords:** non-structural carbohydrates, storage, growth, stress, urban forest, *Celtis occidentalis*, *Fraxinus pennsylvanica*, *Tilia cordata*

## Abstract

Trees that grow in urban areas are confronted with a wide variety of stresses that undermine their long-term survival. These include mechanical damage to the crown, root reduction and stem injury, all of which remove significant parts of plant tissues. The single or combined effects of these stresses generate a complex array of growth and ecophysiological responses that are hard to predict. Here we evaluated the effects of different individual and combined damage on the dynamics of non-structural carbohydrates (NSC, low weight sugars plus starch) concentration and new tissue growth (diameter increment) in young trees. We hypothesized that (i) tissue damage will induce larger reductions in diameter growth than in NSC concentrations and (ii) combinations of stress treatments that minimally alter the “functional equilibrium” (e.g., similar reductions of leaf and root area) would have the least impact on NSC concentrations (although not on growth) helping to maintain tree health and integrity. To test these hypotheses, we set up a manipulative field experiment with 10-year-old trees of common urban species (*Celtis occidentalis, Fraxinus pennsylvanica*, and *Tilia cordata*). These trees were treated with a complete array of mechanical damage combinations at different levels of intensity (i.e., three levels of defoliation and root reduction, and two levels of stem damage). We found that tree growth declined in relation to the total amount of stress inflicted on the trees, i.e., when the combined highest level of stress was applied, but NSC concentrations were either not affected or, in some cases, increased with an increasing level of stress. We did not find a consistent response in concentration of reserves in relation to the combined stress treatments. Therefore, trees appear to reach a new “functional equilibrium” that allows them to adjust their levels of carbohydrate reserves, especially in stems and roots, to meet their metabolic demand under stressful situations. Our results provide a unique insight into the carbon economy of trees facing multiple urban stress conditions in order to better predict long-term tree performance and vitality.

## Introduction

Trees are among the most valuable components of urban green areas due to the wide range of environmental, social, cultural, and economic benefits they provide (Konijnendijk et al., 2005). Nevertheless, urban trees are often facing both biotic and abiotic damage, which affect their health and integrity from canopy to root level. Such damage includes defoliation by insects and wind-breakage, stem damage due to frost or injuries leading to loss of woody tissues and transport capacity (Sieghardt et al., 2005), as well as root damage due to road and house repair and construction (North et al., 2017). Gray infrastructure often limits the growing space of trees, and when combined with compacted soils, water and atmospheric pollution, further exacerbates the stress conditions caused by various damage (Konijnendijk and Randrup, 2004; Tubby and Webber, 2010). Additionally, other damage such as girdling, or ring-barking, often occurs from bicycles chained to street trees, lawn mowers, weed trimmers and human vandalism (Moore, 2013; Purcell, 2014).

Tissue loss changes the growth (the annual change in standing biomass accumulated) and allocation patterns between above- and below-ground tree components, affecting the functional balance of the tree (Quentin et al., 2012; Wiley et al., 2017; Dong et al., 2019). The reduction in photosynthetic tissue leads to a decrease in carbon resources produced, and in photosynthates available for growth and reserve accumulation (Handa et al., 2005; Wiley et al., 2013; Atkinson et al., 2014; Deslauriers et al., 2015). Severe defoliation may even cause root mortality by decreasing metabolic activity (e.g., water and nutrient uptake) (Snyder and Williams, 2003). Root reduction causes a deficiency of water and nutrients reducing photosynthetic metabolism (Dong et al., 2016). Additionally, root reduction removes sink-structures and reduces storage capacity (Landhäusser and Lieffers, 2003). The removal of the bark and cambium through stem damage influences the transport capacity between sources and sinks thus hindering the refilling or mobilization of reserves (Högberg et al., 2001; Moore, 2013; Purcell, 2014; Mei et al., 2015). However, stem damage typically allows respiration as water transport is carried out through the xylem.

New growth to restore the functional balance between above- and below-ground tissues after stress may depend on the amount of carbohydrate reserves in storage pools, as the remobilization of reserves support metabolic functioning during stressful periods and can produce compensatory growth or additional reserve investment facilitating recovery after the stress (Chapin et al., 1990; Dietze et al., 2014; Ramirez et al., 2018). Carbohydrate reserves are mainly comprised of non-structural carbohydrates (NSC) that are formed from low weight sugars and starch (Hoch et al., 2003). Sugars are mobilized easily and used for short-term metabolism, while starch is stored in a more recalcitrant form for long-term use during and after periods of severe stress (Chapin et al., 1990; Dietze et al., 2014; Martínez-Vilalta et al., 2016).

Photoassimilates are allocated to growth or to reserves and other physiological functions such as defense (Chapin et al., 1990; Dietze et al., 2014; Hoch, 2015). Thus, the concentration of NSC in tree tissue depends on the ability of components to use plant-available resources (sink strength), and on the long-distance transport between the carbon sources (either NSC in pools or carbohydrates synthesized by leaves) and carbon sinks (mainly respiratory metabolism, storage of NSC, and tissue growth) (Lacointe, 2000; Minchin and Lacointe, 2005). Therefore, damage that results in tissue loss will affect the carbon allocation priorities. This response will depend both on the functional role of the organs involved and timing of the damage, as different organs may function as carbon sources or carbon sinks at different times (Li et al., 2002).

Concurrent damage can cause urban tree health to decline and, in extreme cases, lead to mortality (Calfapietra et al., 2015). However, the physiological response of trees to a combination of stress-factors is generally unclear (Niinemets and Valladares, 2008; Niinemets, 2010). The interaction of several stress-factors generates a unique response that may be more severe (negative interaction) or less severe (positive interaction) than the sum of their individual effects (Mittler, 2006; Niinemets, 2010). Here, we evaluated the effects of different individual and combined stresses on the dynamics of NSC reserves in tree saplings using a fully-factorial manipulative experiment with three common North American urban tree species (*Fraxinus pennsylvanica*, *Celtis occidentalis*, and *Tilia cordata*). Trees were subjected to increasing levels of commonly occurring urban damage: (1) three levels of defoliation, (2) three levels of root reduction, and (3) two levels of stem damage. These stress treatments were applied individually and in combinations of two or three simultaneously at different intensities. These disturbance treatments offered the potential to act in a complementary way modifying primarily the carbon sources (defoliation), sinks (root reduction), and transport capacity (stem damage). Hence, each treatment created an imbalance between resource production and reserve demand and availability (Farrar and Jones, 2000).

Since plants require a minimum concentration of NSC stored to maintain their basic functioning (McDowell, 2011; Sala et al., 2012; Martínez-Vilalta et al., 2016), we hypothesized that (i) tissue damage will induce larger reductions in growth than in NSC concentrations, and (ii) combinations of stress treatments that minimally alter the “functional equilibrium” (e.g., similar reductions of leaf and root area) will have the least impact on NSC concentrations (although not on tree growth) helping to maintain tree health and integrity.

## Materials and Methods

### Study Site

The study was conducted in the municipal nursery of the city of Montreal, province of Quebec, Canada. The site lies at 45°30″N, 73°33′W (about 35 m of elevation). The mean annual precipitation is 978 mm (215 mm snow and 763 mm rain). The mean annual temperature is 6.2°C and the mean annual growing season temperature is 14.4°C, lasting generally from May to October.

### Study Species

We studied three tree species that are among the most commonly planted trees in the city of Montreal: *Celtis occidentalis* Linnaeus (Common Hackberry; native), *Fraxinus pennsylvanica* Marsh. (Green ash; native), and *Tilia cordata* Mill. (little-leaf linden; introduced in America from Europe). These species have growth strategies and growth rates considered as moderate, intermediate and rapid, respectively, which could determine different responses of reserve allocation and growth under stress (Table 1).

**TABLE 1:** Functional characteristics of the tree species studied.

Species/Trait	*Celtis occidentalis*	*Fraxinus pennsylvanica*	*Tilia cordata*
Foliar carbon (%)	41.0	46.5	47.0
Foliar nitrogen (%)	1.2	2.0	2.7
Foliar carbon/nitrogen	33.9	23.4	18.0
Specific leaf area (mm^2^ mg^–1^)	17.3	15.2	18.6
Photosynthetic capacity (μmol CO_2_ m^–2^ s^–1^)	6.0	13.7	15.3
Wood density (mg mm^–3^)	0.7	0.6	0.4
Growth rate	Moderate	Intermediate	Rapid
Shade tolerance*	Intermediate	Intermediate	Tolerant
Lifespan	Moderate	Short	Moderate

*Data from Niinemets and Valladares (2006).

### Growth Measurements

The trees were field-grown seedling-propagated in 2003 and 2004 in the Montreal municipal nursery. The average diameter at 40 cm above the ground and height of the trees at the beginning of the study (July 2012) were 53.5 (7.8) mm and 3.1 (0.6) m for *C. occidentalis*, 51.8 (8.9) mm and 4.4 (0.6) m for *Fraxinus pennsylvanica*, and 63.5 (4.7) mm and 4.3 (0.3) m for *Tilia cordata* (standard deviation in brackets). The diameter was measured with a Mitutoyo Digimatic caliper (0.01 mm accuracy) at 40 cm above ground level to avoid actively growing branches. To increase accuracy, diameter was measured along two perpendicular axes at a height marked with a permanent steel nail. Tree height was measured with a TruPulse 360 laser with a resolution of 10 cm for linear lengths (Laser Technology, Inc., CO, United States). Trunk diameter (at 40 cm height) and total tree height were measured periodically between July 2012 and May 2015. Diameter was measured approximately every 2 months during the growing season while the height was measured annually. To allow for comparisons between the different tree species, growth measurements were normalized.

### Stress Treatments

At the beginning of the study, trees were assigned randomly to one of 18 treatment combinations (see details below). Treatments were first applied in July 2012 and repeated in July 2013, which corresponded to the month of maximum leaf area. The stress treatments consisted of various gradients of defoliation, root reduction, and stem damage. The total number of trees in the experiment was 267 individuals (116 *C. occidentalis*, 86 *F. pennsylvanica*, and 65 *T. cordata*). Individuals of each species were divided in blocks ensuring at least three trees per treatment given the availability of individuals. Thus, three blocks were established in the case of the *T. cordata*, four blocks for *F. pennsylvanica* and six blocks for *C. occidentalis* (Supplementary Figure 1).

**FIGURE 1:**
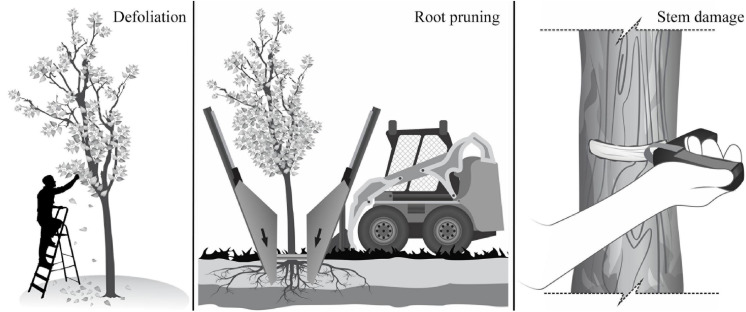
Schematic representation of the stress treatments applied to trees of *F. pennsylvanica*, *C. occidentalis*, and *T. cordata*: defoliation, root reduction, and stem damage.

The experiment was set up as a fully factorial design with three levels of defoliation (DF) [severe (75%), moderate (37%) and control (0%)], three levels of root reduction (RR) [severe (75%), moderate (37%) and control (0%)], two levels of stem damage (SD) [severe (50%) and control (0%)], and all the possible combinations among these three treatments and intensities (Figure 1). The defoliation (DF) treatment consisted of removing leaves manually at the base of the petiole, removing 37% of leaves per branch in the moderate treatment, and 75% in the severe treatment (Figure 1). The root reduction (RR) treatment consisted of removing part of the root system outside a 30 cm radius from the trunk using a tree spade machine (Figure 1). The machine, with independent blades that encircled the tree, cut the roots to a depth of 1.2 m, and removed 37% in the moderate treatment and 75% in the severe treatment. The stem damage (SD) treatment consisted of removing a 40 mm wide band around 50% of the stem circumference at 30 cm above the ground using a bark blaster girdling tool that removed both the cambium and phloem (Figure 1).

### Analysis of Carbohydrate Concentrations

In the spring and autumn of 2014, we measured the concentrations of NSC (low weight sugars plus starch) in roots, stems, and branches of all 267 tree saplings. These measurements took place the growing season after the stress treatments were applied in July 2013 to monitor tree reaction in terms of growth reallocation and reserve utilization. The concentrations of NSC in leaves were only measured in the summer of 2014. Stem samples were taken with a 4.3 mm diameter increment borer at 130 cm from the ground for each individual. Root samples were taken with a 4.3 mm increment borer from large surface roots within 10 cm of the base of the stem. Stem and root samples were comprised of phloem and xylem from all growth rings including the pith but not the outer bark. Top branches were cut with a tree trimmer so that the twenty leaves per tree were sampled from the entire canopy. Collected samples were placed in paper bags and refrigerated right after collection. Within 8 h of collection samples were microwaved in the lab to stop enzymatic activity (Popp et al., 1996), oven dried, and ground using a ball mill. Samples were analyzed for NSC concentration following the standard methods proposed by Hoch et al. (2002). Ground plant material was dissolved for 30 min in distilled water. Starch was broken down into glucose, and sucrose into glucose and fructose with clarase (*Aspergillus oryzae*, Enzyme Solutions Pty Ltd., Croydon South, VIC, Australia) incubation at 40°C for 15 h. Phosphoglucose-isomerase was added to the solution. The total amount of glucose, which corresponded to total NSC, was quantified photometrically in a microplate photometer at 340 nm (Thermo Fisher Scientific, Waltham, MA, United States) after the conversion of glucose to gluconate-6-phosphate (hexokinase; Sigma-Aldrich, St. Louis, MO, United States). Subsequently, an aliquot of the original extract was treated with invertase and phosphoglucose-isomerase (both Sigma-Aldrich) to determine the amount of glucose, fructose, and sucrose with a glucose test (see above). Starch was calculated as NSC minus soluble sugars (soluble sugars = sucrose + fructose + glucose). Pure starch and glucose, fructose, and sucrose solutions were used as standards. Plant powder from peach leaves (Leco, St. Joseph, MI, United States) was included to control the replicability of the extractions. NSC concentrations are reported as percentage of dry matter.

### Statistical Analysis

To avoid autocorrelation of errors produced by continuous measures of diameter and height, and reserve concentrations (soluble sugars, starch, and NSC), linear mixed-effect models were used to evaluate the effect of the stress treatments (DF, RR, and SD) and their interactions on growth and reserve concentrations in the different tissues (leaves, roots, stems, and branches) per species (Gregoire, 1987). Blocks were used as random effect (Supplementary Figure 1). Model calculations were performed using the R package “lme4” (Bates et al., 2014). The function *difflsmeans* in the “lmerTest” package (Kuznetsova et al., 2016) was used as a *post hoc* test to determine differences in the least square means among treatment combinations. The relationship between concentration of reserves and growth was assessed through the coefficient of determination (Pearson’s *r*). All statistical analyses were performed with the software R (R Foundation for Statistical Computing, Vienna, Austria).

## Results

### Effects of Stress on Tree Growth

**FIGURE 2:**
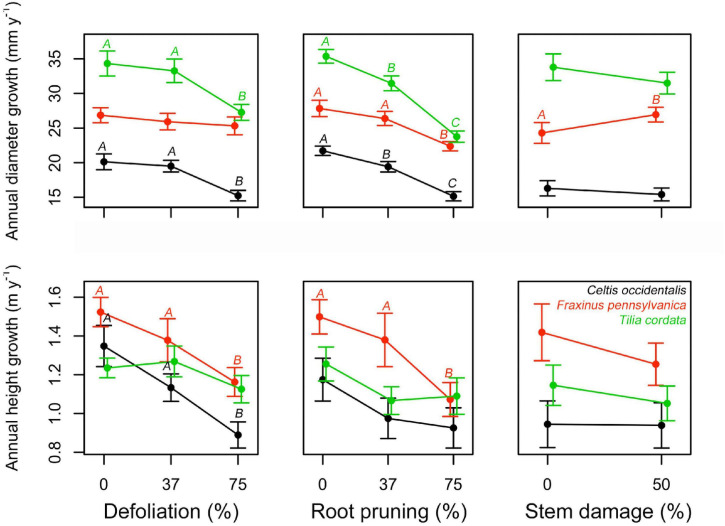
Single effects of a gradient of defoliation, root reduction, and stem damage on diameter and height growth of *Celtis occidentalis*, *Fraxinus pennsylvanica*, and *Tilia cordata*. Error bars represent the standard error of the mean. Different letters represent significant mean differences between stress levels.

No mortality was recorded during the study period. All treatments had a negative impact on both diameter and height normalized growth for all species, except for stem damage in *F. pennsylvanica* that caused a significant increase in diameter growth (Figure 2, Supplementary Figures 2, 3, and Supplementary Table 1). In most of the stress cases, only severe stress had a significant effect on the normalized growth (75% DF or 75% RR), except for *C. occidentalis* and *T. cordata* where there was already a significant effect at low root reduction intensities. Stem damage only significantly affected the diameter growth of *F. pennsylvanica*. Interactions seemed mostly non-significant. Only the interaction between defoliation and root reduction (DF:RR) reduced diameter growth of *C. occidentalis* and the interaction between defoliation and stem damage (DF:SD) reduced height growth on *F. pennsylvanica* (Figure 2, Supplementary Figures 2, 3, and Supplementary Table 1).

### Effects of Single Stress Treatments on NSC Concentrations

**FIGURE 3:**
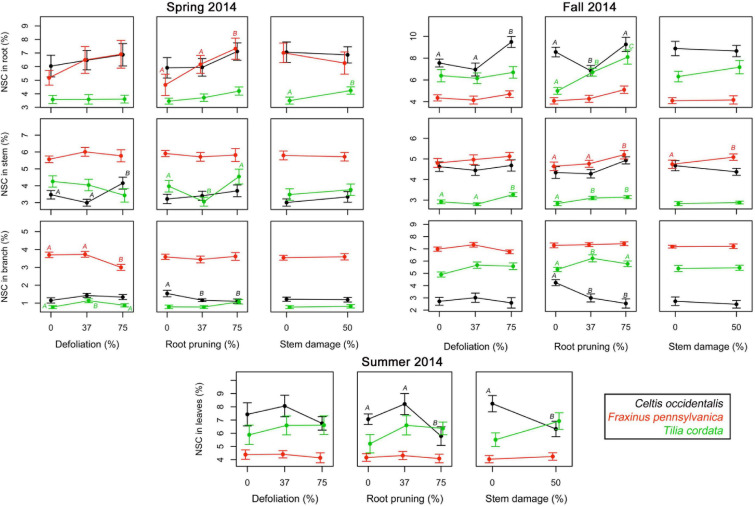
Single effects of a gradient of defoliation, root reduction, and stem damage on non-structural carbohydrates (NSC) concentrations in tissues of *Celtis occidentalis*, *Fraxinus pennsylvanica*, and *Tilia cordata* in the spring, summer and fall of 2014. Error bars represent the standard error of the mean. Different letters represent significant mean differences between stress levels.

In spring 2014, the first measurements of NSC in woody tissues showed concentrations between 0.4 and 12.0% and at the second assessment in fall 2014 concentrations varied between 1.1 and 11.7%, depending on tissues, species, and treatments (Supplementary Figures 4, 5). Concentrations of NSC in leaves measured in summer 2014 varied between 3.3 and 12.7%, with highest concentrations in the control trees of *C. occidentalis* and lowest in *F. pennsylvanica* (Supplementary Figure 6). Reserve concentrations were in most cases not significantly affected by single treatments in neither spring nor autumn measurements (Figure 3 and Supplementary Table 2). When the effects were significant, high intensity treatments tended to significantly increase reserve concentrations in stems and roots in both periods, but reduced their concentration in branches (Figure 3). Single treatments of root reduction and stem damage significantly decreased the NSC concentrations in leaves of *C. occidentalis* only (Figure 3 and Supplementary Table 2).

### Effects of Combined Stress Treatments on NSC Concentrations

The number of significant interactions between stress treatments and reserve concentrations was extremely low (Figure 5 and Supplementary Table 2). In spring 2014, severe defoliation and stem damage interactions were significant and increased NSC concentrations in *C. occidentalis* stems. In *Fraxinus pennsylvanica*, moderate defoliation (37%) along with an increase in stem damage decreased NSC concentrations in roots of *F. pennsylvanica* relative to severe defoliation (75%) with stem damage. In fall 2014, severe root reduction and stem damage had a significant effect and reduced NSC concentrations in roots. The interaction of root reduction and stem damage had a significant negative effect on NSC concentrations in leaves in summer 2014 in both *C. occidentalis* and *F. pennsylvanica*. For *T. cordata* no significant interactions were found.

### Relationship Between NSC Concentrations and Tree Growth

Higher NSC concentrations were consistently associated with lower diameter increments for all three species (Figure 5). Specifically, we found significant negative correlations between diameter increment and NSC concentrations in roots and stems of *C. occidentalis*, roots of *F. pennsylvanica*, and roots, and stems and branches of *T. cordata.* On the contrary, we did not find any significant correlation between height increment and NSC concentrations in any tissues of the three species for both treatment periods (data not shown).

## Discussion

Assessing the impact of different levels of defoliation, root reduction, and stem damage separately and in combinations, we observed a consistent decline in tree diameter and height growth with increasing stress intensity; the sole exception was the positive effect on diameter growth of *F. pennsylvanica* following stem damage (Figure 2 and Supplementary Table 1). However, the impacts of different levels of defoliation, root reduction and stem damage on carbohydrate reserves were (1) less consistent, (2) did not follow a simple linear trend with increasing stress intensities, (3) were relatively mild, and (4) changed with each organ, species and combination of treatments. We found some significant interactions among the different stress treatments that make any interpretation of single effects difficult. Nonetheless, in cases where some effects were observed, single stress treatments induced an increase in reserve concentrations in stem and roots, and a decrease in branches. On the contrary, combined stress treatment effects on reserve concentrations were more variable both among species and the tree organs evaluated.

### Effects of Single Stress Treatments on Tree Growth and Reserve Concentrations

As hypothesized, both diameter and height growth were negatively affected by the increasing level of stress intensity inflicted on the trees (Figure 2 and Supplementary Table 1). At the end of the study, differences in growth between control and the most affected trees were between 13 and 19% for diameter and between 9 and 16% for height (Supplementary Figure 2). Although the growth responses to stress treatments are complex and depend on the species and type of stress applied, our results are quantitatively similar to the growth reduction reported for defoliation and root reduction treatments in other studies (Quentin et al., 2011; Jacquet et al., 2012; Wiley, 2013; Dong et al., 2016). This reduction in growth can be explained by the lower carbon uptake due to defoliation, and lower water and nutrient uptake due to root reduction. Interestingly, although we expected stem damage to induce a decrease in tree growth due to the disruption of the transport system and consequent reduction of photosynthate movement to the roots (Regier et al., 2010; Mei et al., 2015), we instead found a significant increase in tree diameter for *F. pennsylvanica*. The stem damage applied to the trees was relatively mild as it only affected 50% of the circumference. It has been reported that most coniferous species are able to resist up to 25% basal girdling (Filip et al., 2007) while some broadleaf species are able to resist more than 50% girdling (Holmes, 1984; Moore, 2013) or even 100% girdling damage such as in white poplar (Glass, 2011). The increase presented in tree diameter of *F. pennsylvanica* may be due to an accumulation of carbohydrates above the wound zone (Moore, 2013) in reaction to the injury as shown in Vitali et al. (2019).

Most of the single defoliation stress treatments did not lead to a significant reduction in the concentrations of NSC in the different tree tissues (Figure 3). In most cases, root reduction caused an increase in the NSC concentrations in the stem as well as the roots. However, in *C. occidentalis* there was a reduction in the NSC concentrations in branches, especially after root reduction. Reduction of carbohydrate reserves in tree organs has been shown to occur in the weeks following stress caused by defoliation (Palacio et al., 2012; Wiley et al., 2013; Atkinson et al., 2014), and stem girdling, especially in the tissues that mobilize reserves to maintain physiological activity (Mei et al., 2015). Nevertheless, a higher concentration of carbohydrates in stems and roots for all three tree species 9 months after the stress treatments in our study may indicate a fast recovery for tree species following injuries to below- and above-ground tree organs, probably indicating a good resilience of these tree species to these types of injuries.

**FIGURE 4:**
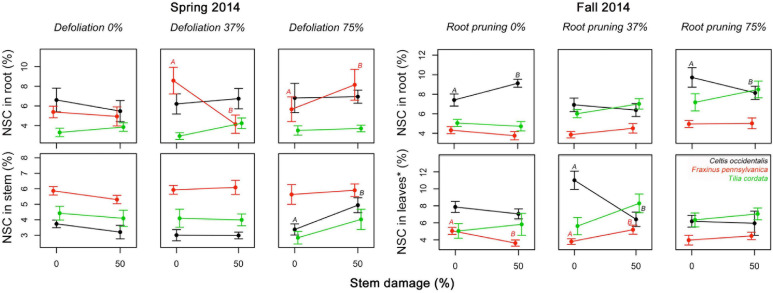
Significant interacting effects of defoliation, root reduction, and stem damage on non-structural carbohydrates (NSC) concentrations in tissues of *Celtis occidentalis*, *Fraxinus pennsylvanica*, and *Tilia cordata* in the spring and fall of 2014. Error bars represent the standard error of the mean. Different letters represent significant mean differences between stress levels.

The increase in carbohydrate reserve concentrations in stems and roots after carbon-limiting conditions may indicate that the stem and roots are secure places to store carbohydrates, and thus they can maintain the availability of resources for resprouting or re-foliation after stress or under possible further stress episodes (Gibon et al., 2009; Wiley, 2013). This carbohydrate increase in these tissues may have been reached through compensatory mechanisms, such as increasing nitrogen concentrations and photosynthetic rates in the remaining foliage after defoliation (Pinkard and Beadle, 1998; Vanderklein and Reich, 1999; Handa et al., 2005; Eyles et al., 2009). Nevertheless, although it is expected that an increase in nitrogen concentrations and photosynthetic rates leads to an increase in carbohydrate concentrations (Li et al., 2016), unlike woody tissues, only a few stress treatments had a significant effect on reserve concentrations in leaves, and those that had a significant effect caused a reduction in the carbohydrate reserves (Figure 4), which suggests no evidence of photosynthetic up-regulation and compensatory responses.

### Effects of Multiple Stress Treatments on Tree Growth and Reserve Concentrations

**FIGURE 5:**
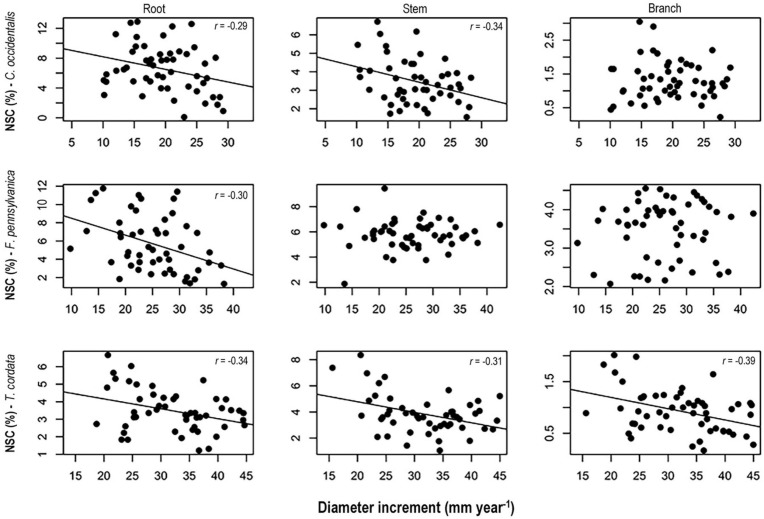
Relationships between non-structural carbohydrates concentrations in spring 2014 and diameter increment in *Celtis occidentalis*, *Fraxinus pennsylvanica*, and *Tilia cordata.* Model lines represent significant relationships between non-structural carbohydrates (NSC) concentrations and diameter increment.

We hypothesized that combinations of stress treatments that alter the “functional equilibrium” the least (e.g., similar reductions of leaf and root area) would have the least impact on NSC concentrations; however, we did not find a consistent response. We expected a positive interaction in reserve concentrations in treatments that involved a combination of defoliation and root reduction compared to single stress treatments. Yet, none of the interactions between DF and RR on reserves were significant (Figure 4 and Supplementary Table 2). We also expected that a combination of tissue loss (by either defoliation or root reduction) and stem damage would lead to a negative interaction (reduction of reserves concentration) because stem damage limits the supply of reserves either to leaves (from roots) or to roots (from new photosynthates). We did find several significant interactions between tissue loss and stem damage but with different patterns. We found that severe defoliation (75%) with stem damage (50%) increased reserve concentrations in roots and stems of *F. pennsylvanica* and *C. occidentalis*, respectively (Figure 4). As suggested above, this may indicate the mobilization of reserves to more secure tissues under severe stress conditions (Gibon et al., 2009; Wiley et al., 2013). However, moderate defoliation (37%) with stem damage (50%) reduced reserve concentrations in roots in *F. pennsylvanica*. Severe root reduction (75%) and stem damage (50%) also caused a reduction in reserve concentrations in roots of *C. occidentalis*, which may indicate that stem damage is limiting the supply of reserves from leaves to roots and thus, roots are spending their reserves in metabolism and/or increasing root production to compensate for root loss and exploit new available soil nutrients and water resources (Mei et al., 2015). Moderate root reduction (37%) and stem damage (50%) caused a contrasting effect in reserve concentrations in leaves of *C. occidentalis* and *F. pennsylvanica* (Figure 5), decreasing NSC in *C. occidentalis* and increasing reserves in *F. pennsylvanica*. A decrease in reserve concentrations after stress treatments in *C. occidentalis* may indicate the lack of compensatory photosynthesis reactions to recover carbon supply (McNaughton, 1983). A reduction in the carbon supply through photosynthesis may lead to a fast depletion of the reserve pools, thus increasing the stress effect on the trees (Niinemets, 2010).

It should be noted that treatments were applied in summer 2012 and 2013, while the NSC concentration measurements were performed the next growing season in spring and autumn of 2014 for woody tissues and in summer for leaves. Sampled leaves were less than 1 year old, branches were generally from the current year and stems and roots were up to several years old. Thus, stem and roots sampled combine pre-treatment, treatment and post-treatment tissues. Since NSC reserves stored in stems and roots serve as a long-term reservoir for responding to high impact damage (Clark and Clark, 1991; Poorter et al., 2010; Clarke et al., 2013) and stay stable over time (Hartmann and Trumbore, 2016), this may be a possible explanation for the lack of a consistent relationship between stress treatments and NSC concentrations in woody tissues. Also, since many models were fitted simultaneously, some of the interactive effects may be spurious due to multiple testing.

### Relationship Between Carbohydrate Reserves and Tree Growth

We found a negative relationship between NSC concentrations and diameter increment suggesting an overall conservative strategy to cope with maintenance respiration, tissue reconstruction or new tissue production following some form of stress (Körner, 2003, 2015; Muller et al., 2011; Figure 5). This relationship was significant in the three woody tissues of *T. cordata* and persistent in roots of all three tree species. This may indicate that trees of *T. cordata* showed higher response to stress than the other two species. Although *T. cordata* is a shade tolerant species, it presents characteristics of fast-growing species such as high foliar nitrogen, photosynthetic capacity, and lower wood density (Table 1). This may indicate a lower allocation of carbon to defense traits and thus a higher dependence on reserves than the other species to maintain a positive carbon balance. These results support the idea that under stress conditions, fast-growing species respond with higher flexibility than slow-growing species (Atkinson et al., 2014).

Our results suggest that following a disturbance to some part of the tree, trees may mobilize accumulated stored NSC over the short term to repair the damage and increase growth, but over the medium to long term the strategy seems to replenish the reserve pool as quickly as possible to the detriment of tree growth. Increasing reserves under the conditions of lower carbon uptake imposed by the stress treatments is consistent with previous studies suggesting that allocation of carbon to reserves is an active process that does not depend on the balance between carbon supply and demand for growth and metabolism; trees regulate the levels of reserves at the expense of growth (Chapin et al., 1990; Silpi et al., 2007; Sala et al., 2012; Wiley and Helliker, 2012). Such behavior in carbohydrate reserves suggests that trees adjust their level of reserves to meet the new metabolic demands (Silpi et al., 2007) because survival under stress conditions may require a higher availability of carbon for maintaining physiological functions, such as metabolism, hydraulic integrity and osmotic exchange of the soluble sugars, instead of maintaining growth (Sala et al., 2012; Wiley and Helliker, 2012). This behavior may be even more marked at increased age (with higher biomass accumulation) and thus, the effect of age should be considered for future analyses since urban forests are mainly composed by mature trees.

## Conclusion

This study examined the single and combined effects of three frequent urban stresses (defoliation, root reduction, and stem damage) on the growth and NSC reserve concentrations in young trees of three common species under field conditions. Our results showed a consistent inverse relationship between diameter growth and total NSC reserve in all three tree species, indicating that trees prioritize reserve accumulation over growth following injuries. Globally, trees tended to accumulate NSC in roots and stems rather than branches 9–12 months following various combinations of stresses. Significant interactions were found between the three stresses applied, indicating that some combinations of stresses while not showing simple additive effects, could modify tree responses. Thus, moderate and mild stress caused by defoliation, root reduction and girdling events do not generally adversely affect plant physiology in terms of growth and reserves. However, trees under severe stress, especially under conditions that alter the functional equilibrium, may drastically reduce their growth rates and, depending on the severity, could eventually die with large amounts of NSC stored in their tissues.

## Data Availability Statement

The raw data supporting the conclusions of this article will be made available by the authors, without undue reservation.

## Author Contributions

JR, IH, and CM contributed to the design of the research. JR carried out the fieldwork. JR performed laboratory analysis with the support CM and IH. JR, VV, and JM-V wrote the manuscript in collaboration with all co-authors. All authors contributed to the article and approved the submitted version.

## Conflict of Interest

The authors declare that the research was conducted in the absence of any commercial or financial relationships that could be construed as a potential conflict of interest.

## Publisher’s Note

All claims expressed in this article are solely those of the authors and do not necessarily represent those of their affiliated organizations, or those of the publisher, the editors and the reviewers. Any product that may be evaluated in this article, or claim that may be made by its manufacturer, is not guaranteed or endorsed by the publisher.
