# Supplementary material for: Reserve Accumulation Is Prioritized Over Growth Following Single or Combined Injuries in Three Common North American Urban Tree Species
Source: Front Plant Sci. 2021 Aug 6;12:715399. doi: 10.3389/fpls.2021.715399 (PMC8378402; doi:10.3389/fpls.2021.715399)

**Supplementary information**

Table S1. Summary of linear mixed model effects showing the effects of defoliation (DF), root reduction (RR), and stem damage (SD) and their interacting effects on diameter and height at the end of the experiment of *Celtis occidentalis*, *Fraxinus pennsylvanica,* and *Tilia cordata* in the fall of 2014. Statistically significant models are shown in bold.

| Growth | | Diameter | | Height | |
| --- | --- | --- | --- | --- | --- |
|  | | F stat | *p*-value | F stat | *p*-value |
| *Celtis occidentalis* | DF | 15.43 | **0.00** | 8.71 | **0.00** |
|  | RR | 34.24 | **0.00** | 1.79 | 0.17 |
|  | SD | 0.12 | 0.73 | 0.05 | 0.83 |
|  | DF:RR | 4.86 | **0.00** | 0.94 | 0.45 |
|  | DF:SD | 0.53 | 0.59 | 0.13 | 0.88 |
|  | RR:SD | 0.26 | 0.77 | 0.84 | 0.43 |
|  | DF:RR:SD | 1.00 | 0.41 | 1.22 | 0.30 |
| *Fraxinus pennsylvanica* | DF | 1.16 | 0.32 | 6.39 | **0.00** |
|  | RR | 12.93 | **0.00** | 5.06 | **0.01** |
|  | SD | 7.03 | **0.01** | 0.09 | 0.76 |
|  | DF:RR | 0.09 | 0.98 | 1.11 | 0.36 |
|  | DF:SD | 0.69 | 0.50 | 4.11 | **0.02** |
|  | RR:SD | 0.06 | 0.94 | 0.68 | 0.51 |
|  | DF:RR:SD | 0.45 | 0.77 | 0.60 | 0.66 |
| *Tilia cordata* | DF | 17.60 | **0.00** | 1.54 | 0.23 |
|  | RR | 59.06 | **0.00** | 1.48 | 0.24 |
|  | SD | 0.55 | 0.46 | 0.05 | 0.81 |
|  | DF:RR | 2.31 | 0.07 | 1.43 | 0.24 |
|  | DF:SD | 0.15 | 0.86 | 0.43 | 0.66 |
|  | RR:SD | 0.10 | 0.90 | 0.49 | 0.62 |
|  | DF:RR:SD | 0.49 | 0.74 | 0.45 | 0.77 |

Table S2. Summary of linear mixed models results showing the effects of defoliation (DF), root reduction (RR), and stem damage (SD) and their interacting effects on NSC concentrations of *Celtis occidentalis*, *Fraxinus pennsylvanica,* and *Tilia cordata* in the spring and fall of 2014. Statistically significant models are shown in bold.

| Tissue | | Root  (Spring 2014) | | Root  (Fall 2014) | | Stem  (Spring 2014) | | Stem  (Fall 2014) | | Branch  (Spring 2014) | | Branch  (Fall 2014) | | Leaves  (Summer 2014) | |
| --- | --- | --- | --- | --- | --- | --- | --- | --- | --- | --- | --- | --- | --- | --- | --- |
|  | | F stat | *p*-value | F stat | *p*-value | F stat | *p*-value | F stat | *p*-value | F stat | *p*-value | F stat | *p*-value | F stat | *p*-value |
| *Celtis occidentalis* | DF | 0.28 | 0.76 | 8.92 | **0.00** | 5.28 | **0.01** | 0.29 | 0.74 | 1.22 | 0.31 | 0.39 | 0.68 | 1.00 | 0.38 |
|  | RR | 0.97 | 0.39 | 6.40 | **0.00** | 0.61 | 0.55 | 2.79 | 0.08 | 3.57 | **0.04** | 6.73 | **0.00** | 4.09 | **0.03** |
|  | SD | 0.04 | 0.85 | 0.18 | 0.67 | 0.90 | 0.35 | 1.14 | 0.29 | 0.13 | 0.72 | 0.32 | 0.58 | 6.19 | **0.02** |
|  | DF:RR | 0.47 | 0.76 | 0.77 | 0.55 | 0.18 | 0.95 | 1.20 | 0.33 | 0.64 | 0.63 | 0.53 | 0.71 | 0.44 | 0.78 |
|  | DF:SD | 0.32 | 0.73 | 1.22 | 0.31 | 3.58 | **0.04** | 0.04 | 0.96 | 1.76 | 0.19 | 1.18 | 0.32 | 0.10 | 0.90 |
|  | RR:SD | 0.58 | 0.56 | 4.01 | **0.02** | 1.69 | 0.20 | 0.09 | 0.91 | 1.14 | 0.33 | 0.57 | 0.57 | 3.27 | **0.05** |
|  | DF:RR:SD | 2.28 | 0.08 | 0.82 | 0.52 | 0.32 | 0.86 | 0.36 | 0.84 | 2.49 | 0.06 | 0.87 | 0.49 | 1.97 | 0.12 |
| *Fraxinus pennsylvanica* | DF | 1.55 | 0.23 | 1.01 | 0.37 | 0.52 | 0.60 | 1.90 | 0.16 | 6.25 | **0.00** | 2.40 | 0.10 | 0.41 | 0.66 |
|  | RR | 3.90 | **0.03** | 2.95 | 0.07 | 0.08 | 0.93 | 6.27 | **0.00** | 0.57 | 0.57 | 0.15 | 0.86 | 0.27 | 0.76 |
|  | SD | 0.89 | 0.35 | 2.00 | 0.66 | 0.03 | 0.85 | 4.49 | **0.04** | 0.00 | 0.95 | 0.02 | 0.88 | 0.10 | 0.75 |
|  | DF:RR | 1.68 | 0.18 | 0.83 | 0.51 | 0.28 | 0.89 | 2.22 | 0.08 | 0.27 | 0.89 | 0.96 | 0.44 | 1.06 | 0.39 |
|  | DF:SD | 5.11 | **0.01** | 0.73 | 0.49 | 0.63 | 0.54 | 0.28 | 0.76 | 2.55 | 0.09 | 0.29 | 0.75 | 0.22 | 0.81 |
|  | RR:SD | 0.36 | 0.70 | 1.23 | 0.30 | 1.77 | 0.18 | 0.08 | 0.93 | 0.95 | 0.40 | 0.35 | 0.71 | 5.80 | **0.01** |
|  | DF:RR:SD | 0.34 | 0.85 | 1.02 | 0.41 | 0.64 | 0.63 | 0.98 | 0.43 | 0.22 | 0.92 | 0.32 | 0.86 | 1.24 | 0.31 |
| *Tilia cordata* | DF | 0.00 | 0.99 | 0.38 | 0.69 | 1.60 | 0.22 | 12.48 | **0.00** | 4.18 | **0.02** | 2.99 | 0.06 | 0.35 | 0.71 |
|  | RR | 2.24 | 0.12 | 11.20 | **0.00** | 4.47 | **0.02** | 6.84 | **0.00** | 3.05 | 0.06 | 3.73 | **0.03** | 1.35 | 0.27 |
|  | SD | 4.41 | **0.04** | 1.69 | 0.20 | 0.41 | 0.53 | 0.38 | 0.54 | 0.18 | 0.67 | 0.04 | 0.85 | 2.92 | 0.10 |
|  | DF:RR | 1.26 | 0.30 | 0.42 | 0.79 | 0.50 | 0.74 | 0.88 | 0.48 | 1.31 | 0.29 | 0.87 | 0.49 | 0.44 | 0.78 |
|  | DF:SD | 0.99 | 0.38 | 0.32 | 0.73 | 1.45 | 0.25 | 0.73 | 0.49 | 0.36 | 0.70 | 0.05 | 0.95 | 0.99 | 0.38 |
|  | RR:SD | 0.67 | 0.52 | 0.99 | 0.38 | 0.38 | 0.69 | 0.23 | 0.80 | 0.58 | 0.56 | 0.57 | 0.57 | 0.63 | 0.54 |
|  | DF:RR:SD | 0.90 | 0.47 | 0.92 | 0.46 | 1.55 | 0.21 | 1.16 | 0.35 | 0.77 | 0.56 | 0.36 | 0.84 | 1.48 | 0.23 |

Fig. S1. Statistical design of the experiment. Left cells correspond to the number of trees per treatment combination in each block. Treatments of: defoliation (DF), root reduction (RR), and stem damage (SD).


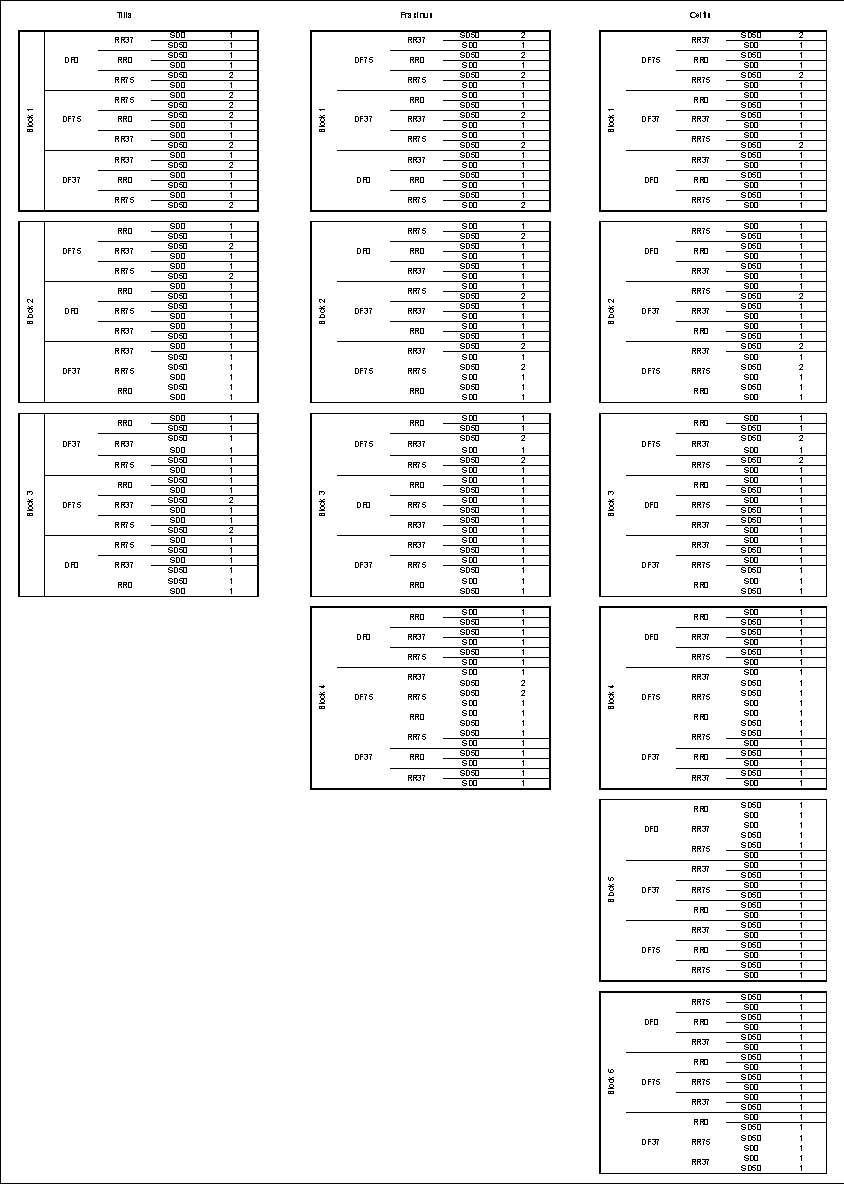


Fig. S2. Diameter and height growth temporal trajectories. Lines show treatment effects of defoliation (DF), root reduction (RR), stem damage (SD) and interactions.


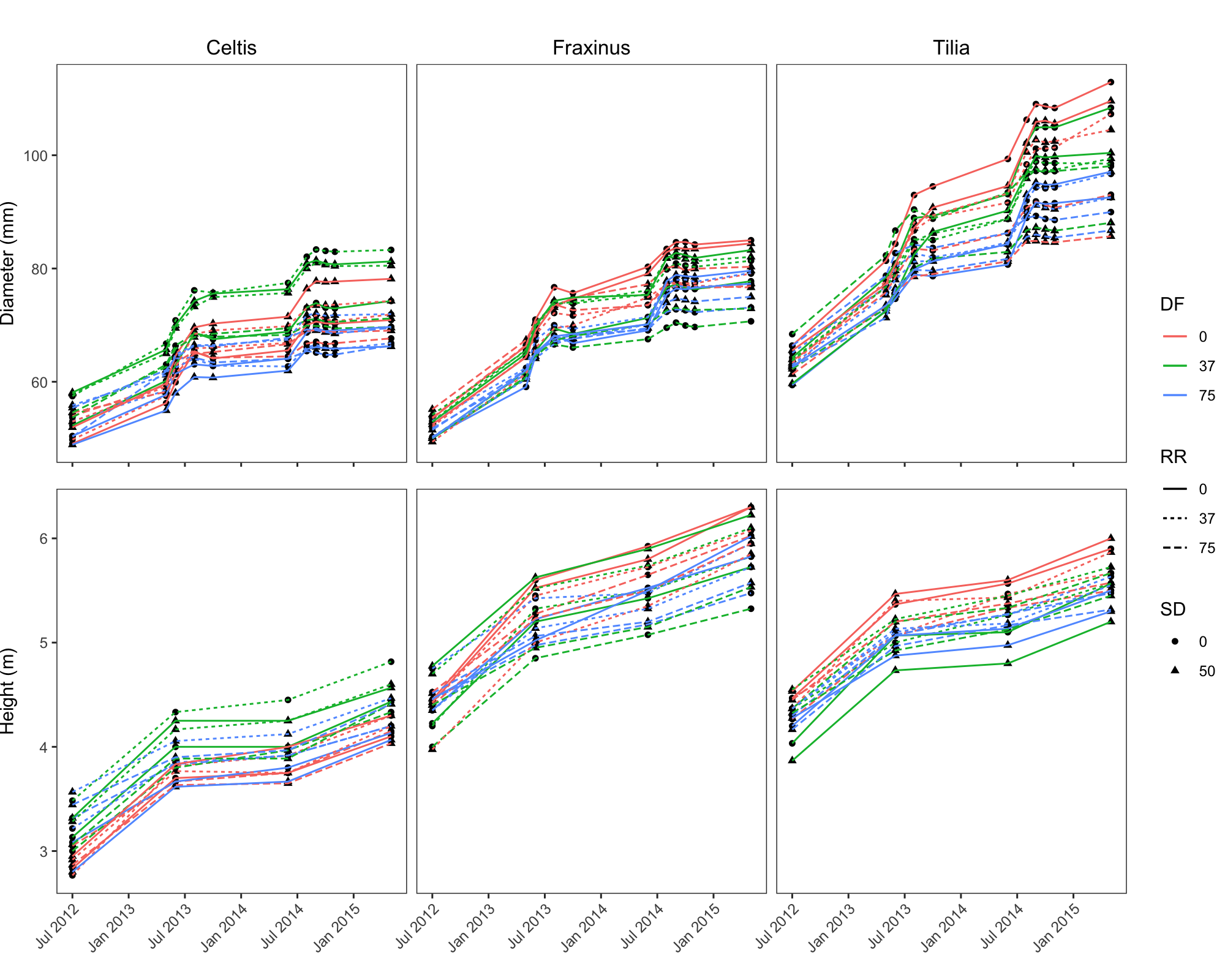


Fig. S3. Influence of both single stress treatments of defoliation (DF, green numbers, on the left), root reduction (RR, red numbers, in the center), and stem damage (SD, blue numbers, on the right) and their combined effects on annual diameter growth of *Celtis occidentalis*, *Fraxinus pennsylvanica*, and *Tilia cordata* in fall 2014. The vertical dashed line corresponds to the average annual diameter growth of the control.


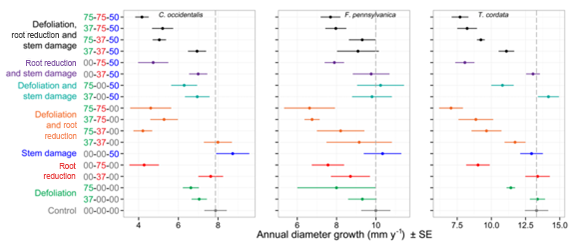


Fig. S4. Influence of both single stress treatments of defoliation (DF, green numbers, on the left), root reduction (RR, red numbers, in the center), and stem damage (SD, blue numbers, on the right) and their combined effects on non-structural carbohydrate (NSC) concentrations of woody organs of *Celtis occidentalis*, *Fraxinus pennsylvanica,* and *Tilia cordata* in spring 2014*.*


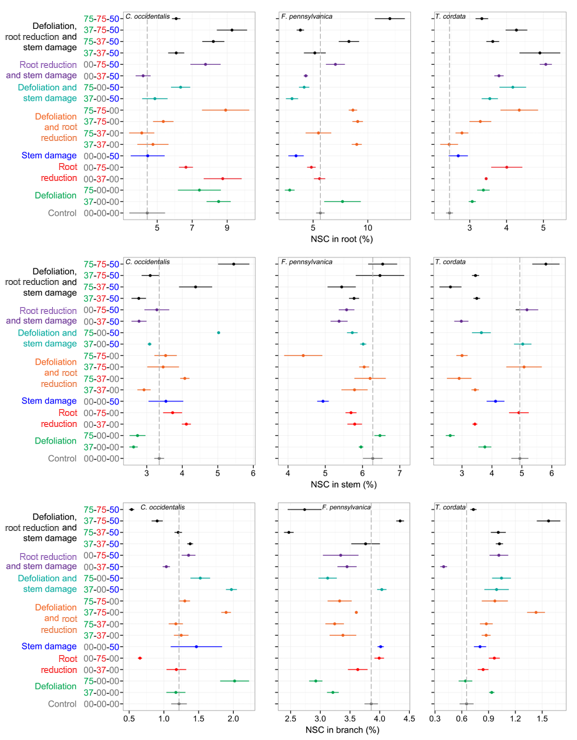


Fig. S5. Influence of both single stress treatments of defoliation (DF, green numbers, on the left), root reduction (RR, red numbers, in the center), and stem damage (SD, blue numbers, on the right) and their combined effects on non-structural carbohydrate (NSC) concentrations of woody organs of *Celtis occidentalis*, *Fraxinus pennsylvanica,* and *Tilia cordata* in fall 2014*.*


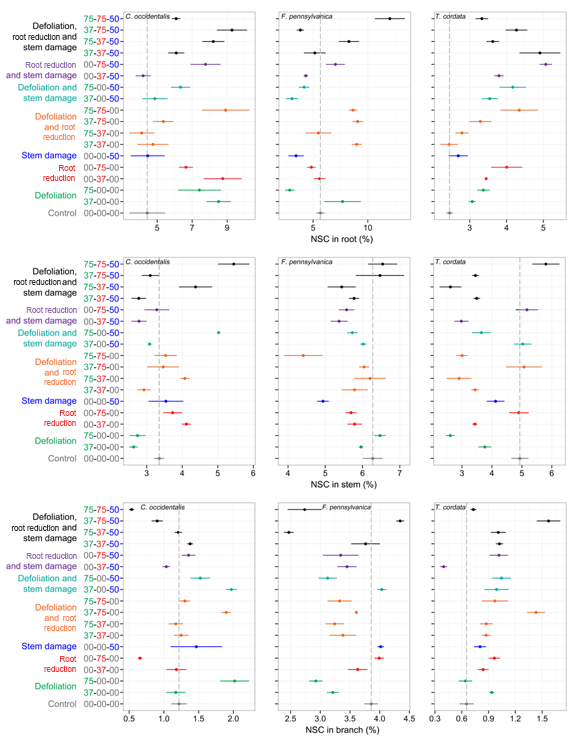


Fig. S6. Influence of both single stress treatments of defoliation (DF, green numbers, on the left), root reduction (RR, red numbers, in the center), and stem damage (SD, blue numbers, on the right) and their combined effects on non-structural carbohydrate (NSC) concentrations of leaves of *Celtis occidentalis*, *Fraxinus pennsylvanica,* and *Tilia cordata* in summer 2014*.*


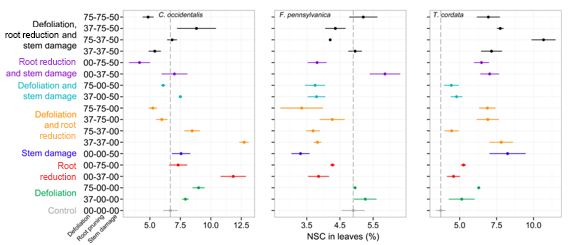

Supplement: Supplementary file 1 [file Data_Sheet_1.docx]
